# Dynamic network properties of the superior temporal gyrus mediate the impact of brain age gap on chronic aphasia severity

**DOI:** 10.1038/s42003-023-05119-z

**Published:** 2023-07-14

**Authors:** Janina Wilmskoetter, Natalie Busby, Xiaosong He, Lorenzo Caciagli, Rebecca Roth, Sigfus Kristinsson, Kathryn A. Davis, Chris Rorden, Dani S. Bassett, Julius Fridriksson, Leonardo Bonilha

**Affiliations:** 1 Department of Health and Rehabilitation Sciences, College of Health Professions, Medical University of South Carolina, Charleston, SC USA; 2 Department of Communication Sciences and Disorders, University of South Carolina, Columbia, SC USA; 3 Department of Psychology, University of Science and Technology of China, Beijing, China; 4 Department of Bioengineering, School of Engineering & Applied Science, University of Pennsylvania, Philadelphia, PA USA; 5 Department of Neurology, Emory University, Atlanta, GA USA; 6 Department of Neurology, Perelman School of Medicine, University of Pennsylvania, Philadelphia, PA USA; 7 Department of Psychology, University of South Carolina, Columbia, SC USA; 8 Department of Electrical and Systems Engineering, School of Engineering & Applied Science, University of Pennsylvania, Philadelphia, PA USA; 9 Department of Physics & Astronomy, School of Arts & Sciences, University of Pennsylvania, Philadelphia, PA USA; 10 Department of Psychiatry, Perelman School of Medicine, University of Pennsylvania, Philadelphia, PA USA; 11 Santa Fe Institute, Santa Fe, New Mexico, NM USA

**Keywords:** Stroke, Cognitive ageing, Network models

## Abstract

Brain structure deteriorates with aging and predisposes an individual to more severe language impairments (aphasia) after a stroke. However, the underlying mechanisms of this relation are not well understood. Here we use an approach to model brain network properties outside the stroke lesion, network controllability, to investigate relations among individualized structural brain connections, brain age, and aphasia severity in 93 participants with chronic post-stroke aphasia. Controlling for the stroke lesion size, we observe that lower average controllability of the posterior superior temporal gyrus (STG) mediates the relation between advanced brain aging and aphasia severity. Lower controllability of the left posterior STG signifies that activity in the left posterior STG is less likely to yield a response in other brain regions due to the topological properties of the structural brain networks. These results indicate that advanced brain aging among individuals with post-stroke aphasia is associated with disruption of dynamic properties of a critical language-related area, the STG, which contributes to worse aphasic symptoms. Because brain aging is variable among individuals with aphasia, our results provide further insight into the mechanisms underlying the variance in clinical trajectories in post-stroke aphasia.

## Abstract (web summary)

Analysis of network controllability in a cohort of 93 participants with chronic post-stroke aphasia suggests that advanced brain aging among these individuals is associated with disrupted superior temporal gyrus dynamics, which in turn contributes to worse aphasia symptoms.

## Introduction

Aphasia is a post-stroke disability defined by impaired language processing. In many individuals, aphasia persists with long-lasting language deficits beyond six months after the stroke (chronic aphasia). However, the interindividual determinants of the severity of chronic aphasia are not well understood. Currently, the location and the size of the stroke lesion are among the most critical predictors of chronic deficits. However, even when considering lesion anatomy combined with baseline severity, only up to 50% of the variance in chronic deficits can be explained^1^. This unexplained variance suggests that other factors play an essential role in chronic language recovery. More specifically, the integrity of the residual brain tissue beyond the lesion is a crucial factor that contributes to the severity and recovery of chronic aphasia^2–7^. Language recovery likely relies on the engagement and adaptation of non-lesioned, non-necrotic residual tissue in language-related or multimodal brain regions. The degree of recovery may depend on the plasticity of the residual tissue, which can be understood as a functional measure of brain health. However, the neurobiological characteristics of the residual tissue that drive recovery, and thus severity in chronic stages of aphasia remain unclear.

The integrity of residual neuronal networks beyond the lesion can be measured using network-based neuroimaging, including structural connectomes constructed from diffusion tensor imaging (DTI) and adapted to evaluate residual networks in peri-lesional and remote areas^8^. Using DTI-based connectivity measures, several groups have demonstrated the importance of link-based integrity (i.e., the preservation of the integrity of the connections between regions or the integrity of white matter pathways) in chronic aphasia^9–15^. Moreover, the topological properties of the residual network also appear to be relevant in predicting long-term deficits. Topological properties refer to the network organization, e.g., which connections are preserved and how the remaining network provides a relational framework between brain structures. For instance, the connections between a pair of regions may be disrupted, but the regions could still be linked by indirect connections^16^. Likewise, brain structures may become segregated (isolated) from the remaining network if hub regions are lost^17–20^. Finally, using the topological properties of networks, it is possible to model information transfer between regions and the dynamic properties of the network^8,21,22^.

In this context, controllability is a promising measure in evaluating the topological integrity of residual brain networks. Controllability is an analytical measurement of a region’s interaction within a dynamic network system and relies on the assumptions of network control theory^23,24^. Network control theory defines different brain states as specific activity levels of each brain region that correspond to specific cognitive functions/domains^24^. Controllability measured at the brain region level quantifies the region’s capability to steer the remaining brain into different states, thereby orchestrating neurophysiological activation patterns at a given time^24–26^. Controllability is estimated from the architecture of the structural white matter network, which determines the region’s structural embedding within the remaining brain network and its ability to distribute input throughout the entire network. The biological relevance of brain network controllability is well document for different conditions^27–32^. Further, recent studies have demonstrated the relation between the controllability of key language-related brain regions and language processing among healthy individuals undergoing brain stimulation and individuals with chronic post-stroke aphasia^21,33^. Because controllability is based on network properties, it correlates with graph theory measures at a node level (e.g., hub status: degree and centrality)^23^. Notably, in our recent work, controllability emerged as the topological brain network feature with the highest explanatory value of post-stroke aphasic symptoms, and outperformed the predictive value of traditional demographic, lesion, and graph-theoretical properties^21^. This is in line with research of other conditions (e.g., epilepsy, psychosis, youth development) also indicating that controllability outperforms graph theory measures^27–31^.

Nonetheless, the relation between topological network properties and other pathophysiological or neurodegenerative factors that influence the severity of aphasia remains poorly understood. More specifically, chronological age (i.e., the age of the stroke survivor) is one of the most consistent and essential predictors of long-term aphasia trajectories^34^. However, to our knowledge, the effects of age on the structural brain network architecture of individuals with aphasia have not yet been established.

Aging effects on brain structure and function are well documented. Age-related structural brain changes are manifested by atrophy, an increase in cerebrovascular fluid spaces, and a decrease in gray matter volume^35,36^. These volumetric changes are especially prevalent in late adulthood after the sixth decade of life, when the slope for cerebrovascular fluid space volume increase is steepest^37^. From a topological point of view, older age is associated with lower connectivity and efficiency within networks^38^ and lower rich club organization^39^ (what refers to a subset of nodes in the network that are densely connected among themselves)^40^. Interestingly, the magnitude of these effects does not occur homogenously across the brain but instead seems to be region dependent. In addition to region-specific variances of aging effects, age-related changes in brain tissue differ across individuals^35,37,41^, which may partially explain variations in individual stroke recovery. A modern concept, brain age, allows for the estimation of age-related changes specific to brain tissue volume that are decoupled from chronological age^42^. The quantification of brain age has been the focus of recent advances in neuroimaging, which predict age from brain tissue. An individual’s biological brain age may be older (or younger) than their chronological age. While brain age is increasingly recognized as a meaningful marker related to cognitive performance among adults^43–45^, less is known about its importance in aphasia and, within the context of this study, its relation to the topological properties of the residual neuronal networks.

Our group recently tested the importance of age-related brain changes in individuals with chronic post-stroke aphasia. The severity of chronic aphasia was predicted by the percentage of preserved long-range white matter fibers, which deteriorate in the presence of age-related neuroimaging findings such as white matter hyperintensities, a common sequela of small vessel brain disease^46^. Moreover, individuals with advanced brain age (i.e., older brain age than chronological age) present more severe language impairments after stroke than those with typical brain age^47,48^. Identifying the underlying neural processes associated with aging in post-stroke aphasia is of high clinical importance because stroke survivors are particularly negatively affected by aging. For example, stroke is more common in older people than in young people; moreover, older people typically have more severe impairments following a stroke, and less recovery compared to their younger counterparts.

To date, the association between age-related changes in structural connectivity and brain function remains largely elusive^49^. However, understanding the association is crucial to explain behavior and performance after brain injury, such as stroke. The rationale for this study was to provide a comprehensive assessment of the relation between tissue loss, age-related effects, impact on networks, and the resulting behavioral effects. A unique opportunity to assess how brain aging impacts brain structure and function is network controllability which measures the influence of specific brain regions on whole-brain network dynamics. The theoretical foundation of network controllability combines the proposed primary effects of aging on brain networks by simultaneously accounting for (1) wide-spread whole-brain network effects, (2) brain-region-specific differences, and (3) structural networks being intertwined with functional networks. Therefore, we sought to test the relation between brain age, regional controllability of language-related regions, and chronic post-stroke aphasia severity. We hypothesized that the average controllability of language-specific regions would explain the relation between advanced brain age and aphasia severity. Our results revealed a link between advanced brain aging, dynamic properties of the superior temporal gyrus (STG), and chronic aphasia severity. These findings suggest that the STG exerts a crucial global network influence contributing to chronic aphasia severity.

## Results

### Brain age

**Fig. 1:**
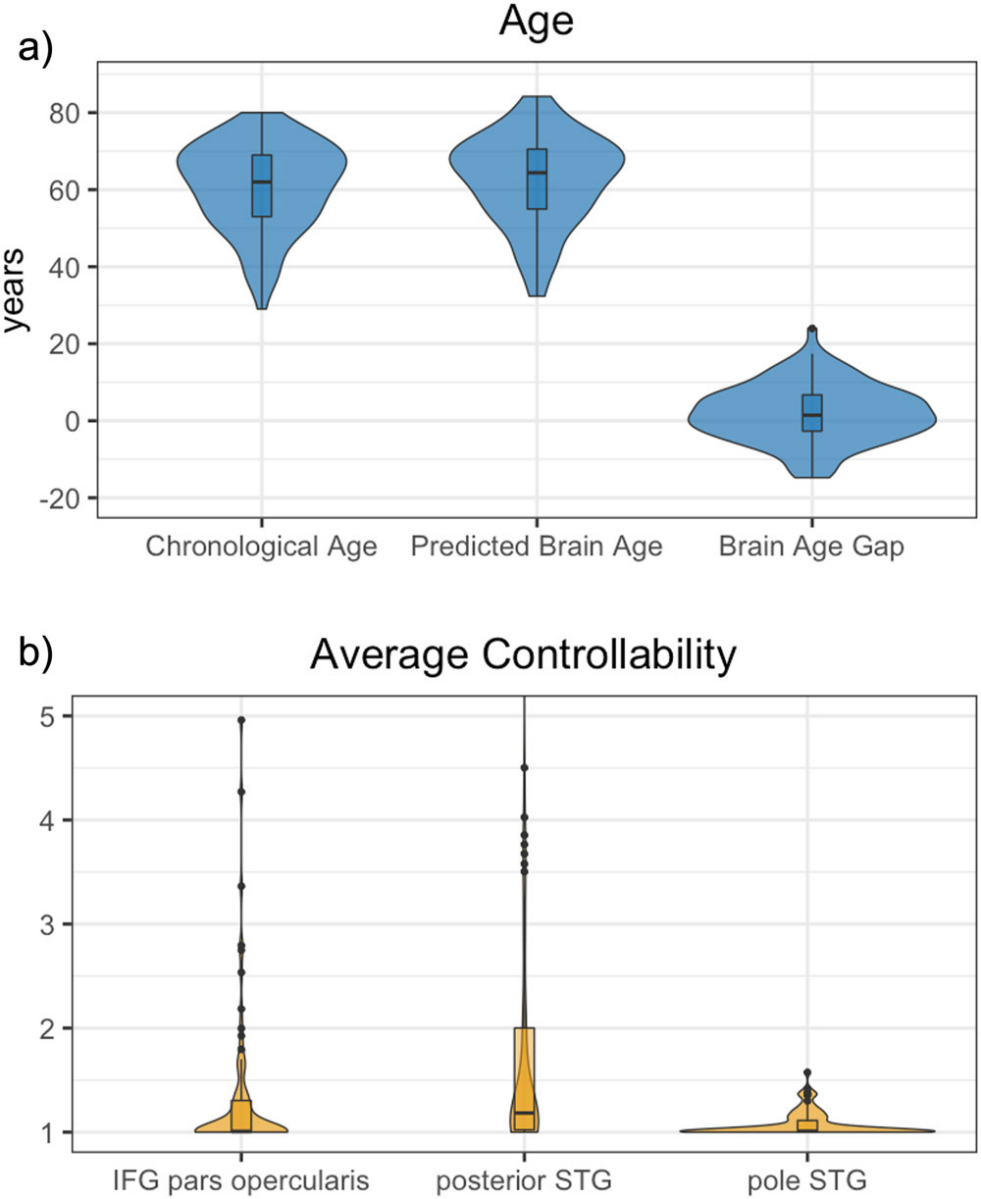
Age and average controllability measures for all *n* = 93 participants. **a** Chronological age, predicted brain age, and brain age gap. **b** Average controllability for the inferior frontal gyrus (IFG) pars *opercularis*, posterior superior temporal gyrus (posterior STG), and the pole of the superior temporal gyrus (pole STG). The y-axis in panel (**b**) was capped at 5 for visualization purposes. Explanation of boxplots: The box represents the interquartile range (first to third quartile), the horizontal line in the box represents the median, and the vertical lines outside the box (whiskers) represent data points outside the interquartile range.

Participants (*n* = 93) were on average 60.77 years old (*SD* = 11.19, median = 62.00), and the predicted brain age was on average 62.51 years (*SD* = 11.56, median = 64.39). Pearson’s correlation between chronological age and predicted brain age was *r* = 0.784, (*p* < 0.001, *r*^*2*^ = 0.615) and the mean absolute error was 6.06 years. The brain age gap was on average +1.95 years (*SD* = 7.46, median = 1.43) (Fig. 1a). Chronological age was significantly lower than predicted brain age (paired Samples T-Test, *t*_(92)_ = −2.51, *p* = 0.014).

### Global controllability

**Fig. 2:**
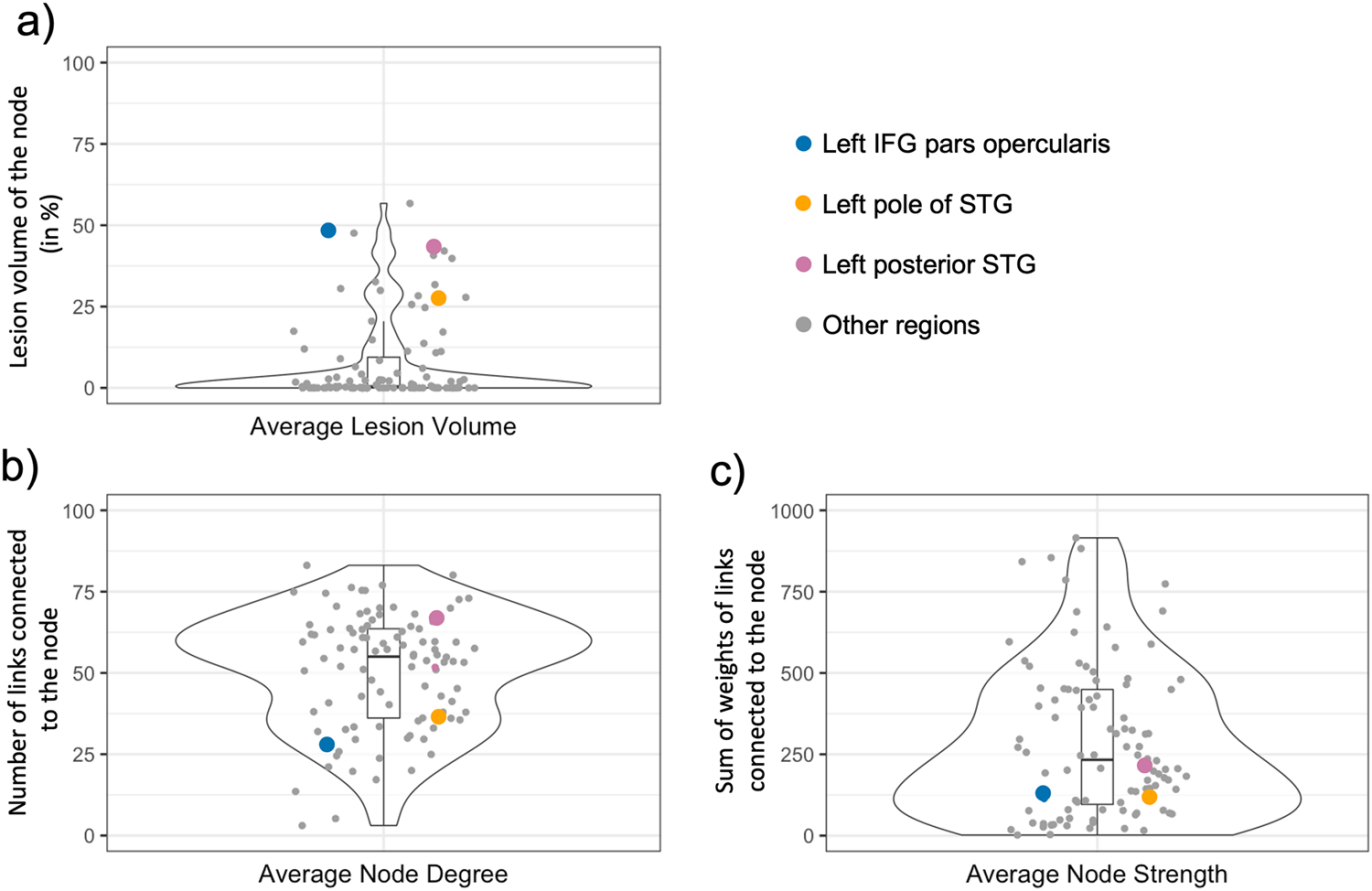
Average regional lesion volume and connectivity measures for each of the 100 gray matter regions (nodes) across all *n* = 93 participants. The inferior frontal gyrus (IFG) pars *opercularis*, posterior superior temporal gyrus (posterior STG), and the pole of the superior temporal gyrus (pole of STG) are highlighted in blue, orange, and pink color, respectively. **a** Average lesion volume of each node in percent. **b** Average node degree for each node which is the number of links connected to the node. **c** Average node strength for each node which is the sum of weighted links connected to the node The y-axis in panel (**b**) was capped at 5 for visualization purposes. Explanation of boxplots: The box represents the interquartile range (first to third quartile), the horizontal line in the box represents the median, and the vertical lines outside the box (whiskers) represent data points outside the interquartile range.

After excluding zero-degree nodes from each participant’s connectome, all participants had controllable brain networks (smallest eigenvalues of the controllability Gramians >0). Across participants, on average one region (median: 0, range: 0–14) was a zero-degree node and thus, excluded from participants’ connectomes. The number of zero-degree nodes significantly correlated with the total lesion volume (Pearson’s *r* = 0.609, *p* < 0.001). The left mamillary body was the most common zero-degree node across all participants (*n* = 15) followed by the right mamillary body (*n* = 12) and the left IFG pars triangularis (*n* = 11). Among all 100 gray matter regions, the mammillary bodies had the lowest number of connections to other gray matter regions. The left mammillary body was connected to on average five, and the right mammillary body to three, other regions across all 93 participants. The left IFG pars triangularis was one of the most commonly lesioned regions with an average of 49% of the region being lesioned across participants (see Supplementary Figure 1 for an overview of the regional lesion volume for left IFG pars *opercularis*, STG pole, and posterior STG). Figure 2a–c shows the average lesion volume, node degree, and node strength for all 100 gray matter regions. Node degree and node strength were calculated using the Brain Connectivity Toolbox^50^ and provide insight into the direct pairwise connectivity of one node with other nodes. Compared to the left posterior STG and left pole STG, the IFG pars *opercularis* had on average a higher regional lesion volume, lower node degree, lower and node strength, thus, the IFG had fewer connections to other regions within the network.

### Average controllability of the language regions of interest

For seven participants, the IFG *pars opercularis* was a zero-degree node and excluded from these participants’ connectomes; the STG pole was a zero-degree node in three participants, and the posterior STG was a zero-degree node in one participant. The average controllability for these three nodes ranged from 1 to 19.25 (IFG *pars opercularis*: 1.00 to 4.96, STG pole: 1 to 1.58, posterior STG: 1 to 19.25) (Fig. 1b). The regional lesion volume significantly correlated with the node’s average controllability for the IFG *pars opercularis* (Pearson’s *r* = −0.297, *p* = 0.005), and STG pole (Pearson’s *r* = −0.357, *p* < 0.001). The correlation between the regional lesion volume and average controllability of the posterior STG was not significant (Pearson’s *r* = −0.180, *p* = 0.087).

### Relation between brain age and aphasia severity

We performed two separate multiple linear regression models to test if chronological age or brain age better predicted WAB-AQ while controlling for stroke lesion volume, time since stroke, education, and sex. Both regression models were significant (*p* < 0.001). The regression model with chronological age as the independent variable explained 29.0% of the variance in WAB-AQ (*F*_(5,85)_ = 6.93, *p* < 0.001, *r*^2^ = 0.290). Chronological age and lesion volume both significantly predicted WAB-AQ (standardized *β* = −0.22, *p* = 0.019, and standardized *β* = −0.52, *p* < 0.001, respectively). The second regression model with brain age as the independent variable significantly predicted 33.3% of the variance in WAB-AQ (*p* < 0.001) (*F*_(5,84)_ = 8.39, *p* < 0.001, *r*^2^ = 0.333). Brain age and lesion volume both significantly predicted WAB-AQ (standardized *β* = −0.30, *p* = 0.001, and standardized *β* = −0.53, *p* < 0.001, respectively).

We then tested if the brain age gap predicted WAB-AQ while controlling for chronological age, lesion volume, time since stroke, education, and sex. The regression model was significant and explained 33.4% of the variance in WAB-AQ (*F*_(6,83)_ = 6.94, *p* < 0.001, *r*^2^ = 0.334). The brain age gap significantly predicted WAB-AQ (standardized *β* = −0.224, *p* = 0.021) independently of chronological age and total lesion volume.

### Relation between brain age gap, controllability, and aphasia severity

**Fig. 3:**
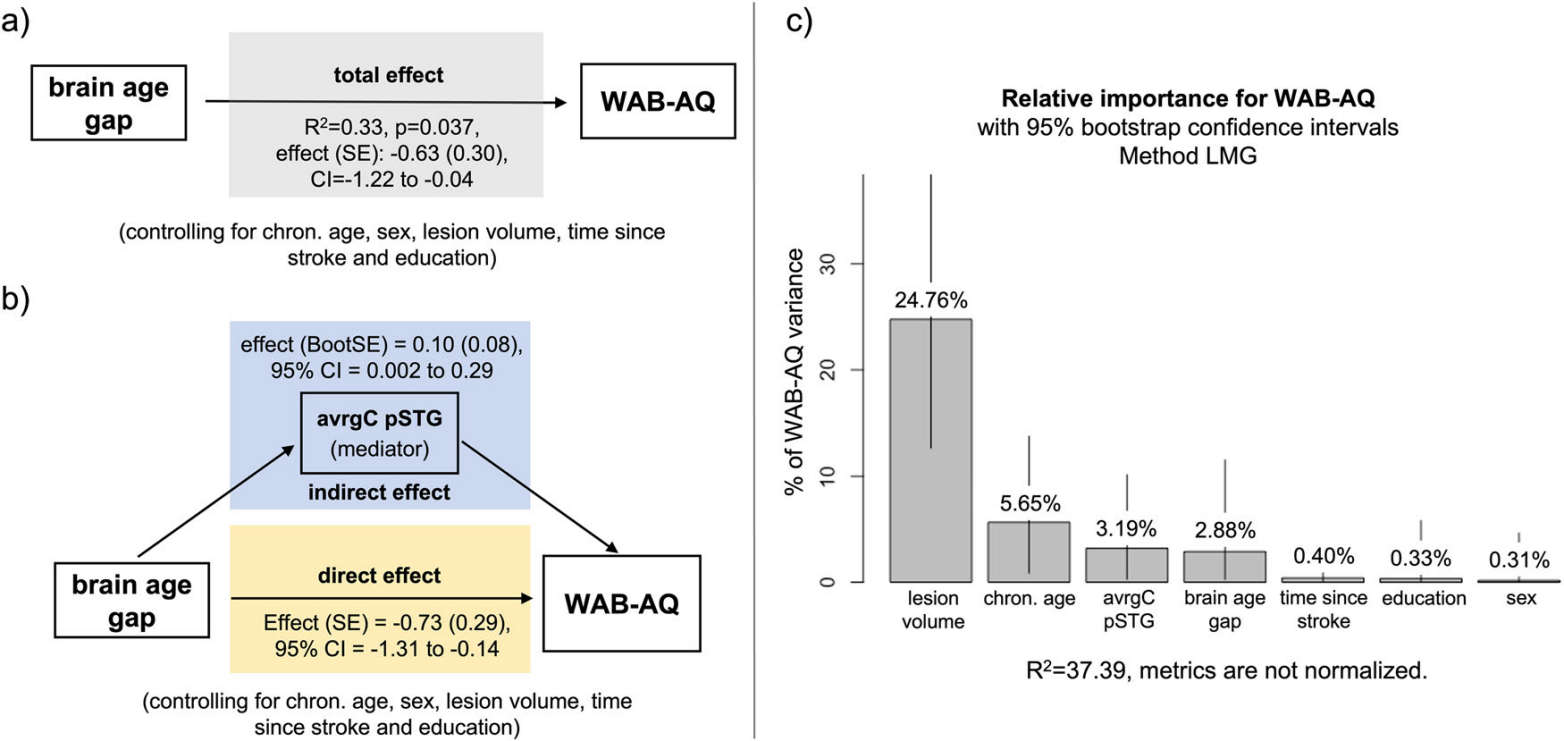
Explanatory power of average controllability. **a** A total effect was observed for the brain age gap on WAB-AQ (Western Aphasia Battery - Aphasia Quotient). **b** A significant direct (yellow box) and indirect effect (blue box) mediated through the average controllability of the posterior superior temporal gyrus (avrgC pSTG) were observed (bootstrapping 95% confidence interval (CI) did not include zero). **c** The independent variables lesion volume, chronological age, brain age gap, and average controllability of the posterior superior temporal gyrus explained together 35.88% of the variance in WAB-AQ. The graph shows the relative importance of each variable. Error bars represent the 95% bootstrap confidence intervals. AvrgC pSTG average controllability of the posterior superior temporal gyrus, chron. age chronological age at time of assessment, LMG Lindemann, Merenda and Gold indices, SE standard error.

Because the brain age gap significantly predicted WAB-AQ, a total effect was established (Fig. 3a), which allowed us to test direct and indirect effects through mediation analysis. We tested the average controllability of the IFG *pars opercularis*, STG pole, and posterior STG as potential mediators. Results indicated a significant direct effect of the brain age gap on WAB-AQ (effect = −0.71, standard error (SE) = 0.29, 95% confidence interval (CI) = −1.28 to −0.14, *p* = 0.013), as well as significant indirect effects of the brain age gap on WAB-AQ mediated by the average controllability of the posterior STG (effect = 0.10, bootstrapping SE = 0.07, 95% CI = 0.001 to 0.27) (Fig. 3b). The brain age gap was associated with the average controllability of the posterior STG, and in turn average controllability of the posterior STG was associated with WAB-AQ scores.

The brain age gap, average controllability of the posterior STG, chronological age, and lesion volume were all significant predictors of WAB-AQ and together explained 35.88% of the variance of WAB-AQ (Fig. 3c). Calculating the relative importance of each regressor for the WAB-AQ, we found that the total lesion volume explained the largest portion of the WAB-AQ variance (24.42%), followed by chronological age (5.49%), average controllability of the posterior STG (3.05%), and the brain age gap (2.91%).

### Post hoc analyses with graph theory measures and lesion volume

We conducted post hoc analyses to explore the relation between the average controllability of the posterior STG and traditional graph theory measures. Using the Brain Connectivity Toolbox^50^, we calculated posterior STG nodal degree, betweenness centrality, and clustering coefficient (metrics to quantify a region’s hub status). In partial correlations that controlled for total lesion volume, we observed significant relations among higher average controllability of the posterior STG and higher nodal degree (Pearson’s *r* = 0.483, *p* < 0.001), higher betweenness centrality (Pearson’s *r* = 0.925, *p* < 0.001), and higher clustering coefficient of the posterior STG (Pearson’s *r* = 0.552, *p* < 0.001).

Further, we tested the relation between the average controllability of the posterior STG and stroke lesion volume and location. The average controllability of the posterior STG did not correlate with the total lesion volume (Pearson’s *r* = −0.085, *p* = 0.421), but showed a trend towards significant correlation with the regional lesion volume of the posterior STG (Pearson’s *r* = −0.180, *p* = 0.087).

**Fig. 4:**
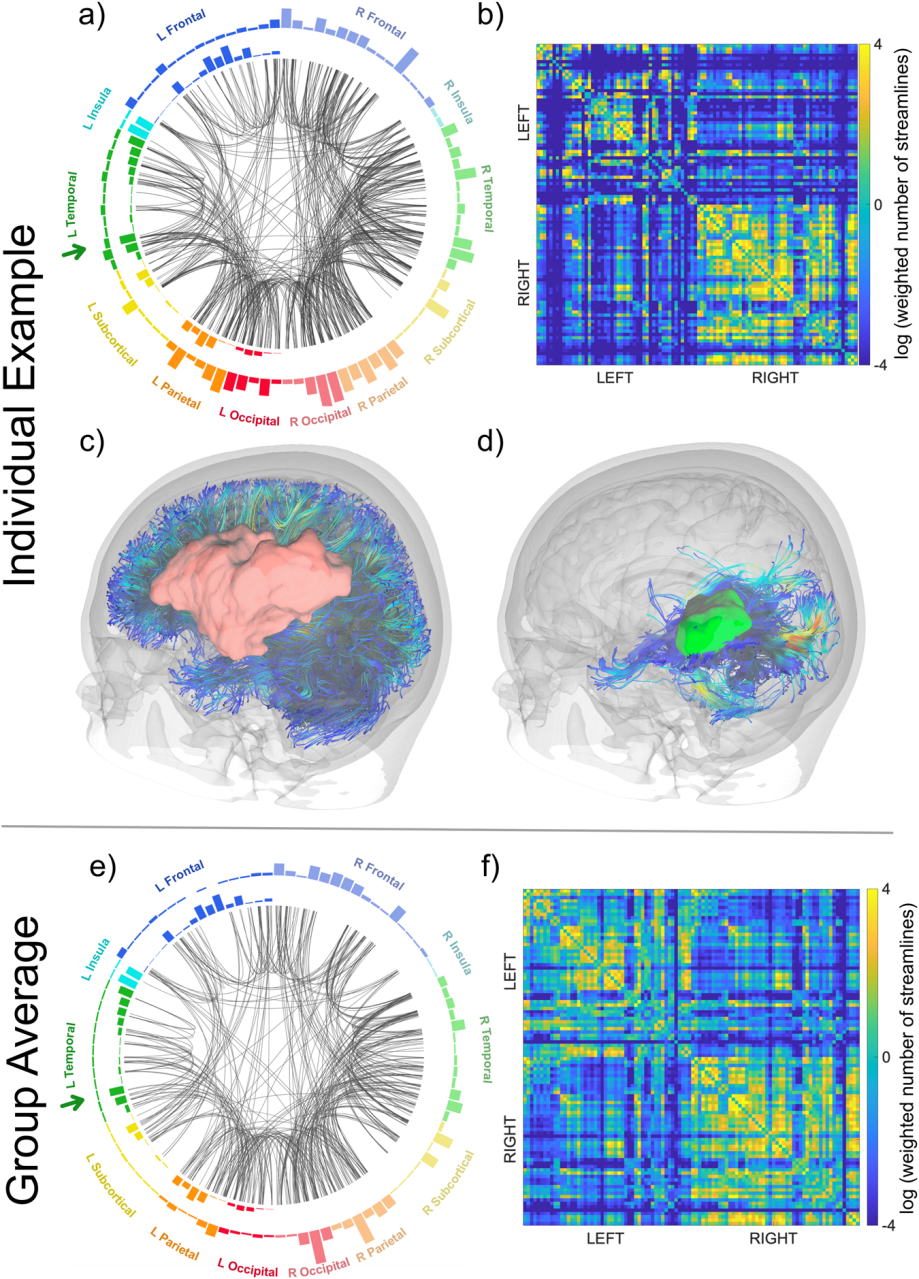
Structural brain connectomes. The upper panel (above the horizontal gray line) presents data from one participant as an example of individual data. The lower panel (below the horizontal gray line) presents group average data. The circular figures of panels (**a**) and (**e**) contain all 100 gray matter regions with left regions on the left and right regions on the right side of the circle (please see Supplementary Table 1 for the position of each region within the circle). The green arrows point to the posterior superior temporal gyrus. The height of the rectangles of the outmost ring shows the average controllability for each region, with taller rectangles indicating larger average controllability. The second ring’s rectangles represent each region’s percentage lesion volume. The streamlines in the inner circle represent the most common (90th percentile) pair-wise connections between the 100 gray matter regions. The heatmaps in panels (**b**) and (**f**) represent adjacency matrices for the probabilistic streamlines between pairs of regions (for better visualization color bars represent log values of the weighted number of streamlines connecting each region pair, from −4 to 4). Panels **c** and **d** show white matter fibers reconstructed from deterministic tractography, for visualization purposes; analyses were performed following probabilistic tractography. The red area in panel (**c**) represents the stroke lesion, and the green area in panel (**d**) represents the posterior superior temporal gyrus.

Figure 4 demonstrates the effects of the stroke lesion on brain connectivity patterns in our study cohort. Due to the stroke locations, the left hemisphere had fewer pair-wise connections than the right hemisphere (Fig. 4a, b, e, f). Thus, the influence of the posterior STG on the remaining brain network depends on (1) connections that survived despite the stroke lesion (Fig. 4c, d) and (2) connections that survived despite the aging process.

## Discussion

Our study evidences a relation among advanced aging (refined through brain tissue-specific aging estimates), structural network architecture, and chronic language deficits in individuals with aphasia. More specifically, we observed that the relation between a higher brain age gap and worse aphasia was mediated by lower controllability of the left posterior STG. Lower controllability of the left posterior STG signifies that activity in the left posterior STG is less likely to yield a response in other brain regions due to the mathematical combination of the properties of the direct and indirect connections of the left posterior STG to the other regions of the network.

Effects of aging on brain network topology are documented in prior research. Aging appears to trigger a reorganization of the brain network with a decrease in connectivity within cortical hemispheres and an increase in connectivity between hemispheres^51^. Further, aging relates to a decrease in brain network integration mirrored by a decrease in the number of hub regions^51^. Hub regions link network communities and allow efficient information to spread between communities and the whole-brain network^19^. Hub regions typically have high average controllability^23^. These findings align with our results concerning the relation between advanced brain aging and reduced average controllability of the posterior STG. The posterior STG has been identified as a hub region within the language network and a key brain area for aphasia recovery in chronic stroke^14^. Through post hoc analyses using graph theory measures, we confirmed that the controllability of the STG is a measure of its hub status. Our results suggest that aging affects the way a critical language hub region, the STG, is embedded within the remaining network, and hence its potential to alter the flow of activity dynamics within the wider brain system. In turn, the changes of activity flow through the STG influence the severity of chronic aphasia.

Among the left posterior STG, STG pole, and IFG *pars opercularis*, the posterior STG was the only region that significantly mediated the relation between the brain age gap and WAB-AQ. Compared to the left STG pole, and IFG *pars opercularis*, the left posterior STG had on average higher node degree and node strength. Thus, the posterior STG was connected to more regions and had more influence on the remaining nodes to distribute information through the network. The importance of preserved structural connectivity in the temporal lobe is well documented. For example, the betweenness centrality of the temporal lobe is a significant predictor of treatment response for individuals with anomia^7^. More specifically, the posterior STG and posterior superior temporal sulcus (STS) have been commonly indicated as major brain areas that contribute to aphasia after stroke. Of note, we segmented the posterior STG based on the JHU brain anatomical atlas, which does not differentiate between gyri and sulci. The segmented brain area labeled as the posterior STG also includes the posterior part of the STS. Damage to areas in the vicinity of the STS has been linked to persistent aphasia^52^. The STS/STG are part of the temporoparietal network, the origin of both major language processing streams—the ventral and dorsal streams^53,54^. Our results suggest that the posterior STG may be a promising treatment target due to its crucial influence on the remaining brain network in people with stroke.

Beyond brain aging, the stroke lesion also impacts the residual brain topology, e.g., through diaschisis effects. We documented significant stroke and aging effects on aphasia severity and thus, provided insight into the cumulative effects of different sources of brain degeneration. The average lesion volume for the participants in our study was 130 ml. Thus, it is unsurprising that the total lesion volume was a significant predictor of aphasia severity, explaining 24% of the variability in the WAB-AQ. Even though we evaluated stroke lesions and brain aging as two independent phenomena, they have shared pathophysiological bases. For example, cardiovascular risk factors (e.g., hypertension, diabetes, smoking) and small vessel brain disease co-occur in people with stroke lesions and advanced brain aging. Our group has shown in previous research that cardiovascular risk factors affect brain network topology independently of a stroke^55^. Compared to individuals without cardiovascular risk factors, those with cardiovascular risk factors have less dense connectomes and a loss of medium and long-range fibers. Cardiovascular risk factors are also associated with small vessel brain disease, a marker for brain health decline among persons with and without stroke^46,56–58^. In recent work, our group tested the relation between advanced brain aging and white matter hyperintensities^59^, commonly recognized as the hallmark neuroimaging marker of small vessel brain disease^60^. A higher load of white matter hyperintensities were related to more advanced brain age. Because white matter hyperintensities are linked to cardiovascular risk factors and are predictors of new or recurrent ischemic strokes^61,62^, monitoring cardiovascular risk factors may be beneficial for delaying brain aging before and after a stroke.

This study has limitations. DTI-derived tractography approximates but does not fully reflect the underlying biology. DTI cannot discern between intra- and extra-axonal water because it relies on signals stemming from a mixture of both. DTI is based on an orientation distribution function, which reflects the preferred direction of water diffusion^63,64^. Consequently, DTI may underestimate the number fibers and may not be able to resolve fiber crossings^65^. We used probabilistic tractography to overcome limitations in DTI estimation of fiber crossing or complex fiber anatomy, but it should be recognized that DTI-derived tractography may have limited accuracy in quantifying connections. Further, we limited our analyses to a pre-defined set of regions of interest to constrain our findings and to conduct hypotheses-driven analyses. Including a larger set of brain regions may have revealed additional significant brain-symptom relationships. Similarly, future studies may use separate language domains as dependent variables to provide a more fine-grained assessment of language performance than the WAB-AQ.

Several algorithms exist to estimate an individual’s brain age^66^. These algorithms differ in their input (structural, functional brain scans) and prediction accuracy (predicted chronological age based on brain data). However, to our knowledge, none of these algorithms have been applied to stroke, and our group is the first to adapt an existing algorithm to stroke lesioned brains. The algorithm by Cole et al. was estimated from a large cohort of healthy individuals and then applied to various disease populations revealing brain age as a predictive marker for disease progression (e.g., in Alzheimer’s, HIV)^42,67–69^. Moreover, prior research found that traumatic brain injury (TBI) accelerates age-related neurodegenerative changes and the brain age gap^70^, relatedly, the brain age gap increases with time since TBI, indicating that the brain injury has worsening consequences over time and may explain cognitive decline with aging in this population. This prior evidence on TBI encouraged the application of the pipeline by Cole et al. in our study of participants with brain injury through stroke. Future research may address individual changes in brain aging caused by stroke-specific and stroke-unspecific factors through longitudinal studies of individuals with stroke and studies of healthy individuals. Those studies may also help to elucidate other factors explaining the relationship between age-related brain structural changes and aphasia severity. While our study demonstrated that the lower controllability of the left superior temporal gyrus is a significant mediator between brain age gap and WAB-AQ, the effect size was rather small, suggesting that other factors are contributing to the relationship.

To conclude, in this study, we identified a link between advanced brain aging, dynamic properties of a critical language region (STG) based on structural network topology and inferred activity dynamics, and severity of chronic aphasia. We demonstrated how the STG exerts a crucial global network influence contributing to chronic aphasia severity and how age-related brain changes can affect individualized trajectories in chronic aphasia. These results corroborate the importance of residual brain tissue beyond the lesion in chronic aphasia severity and suggest that the role of STG controllability in recovery and plasticity is an important direction for future research.

## Methods

### Study participants

**Fig. 5:**
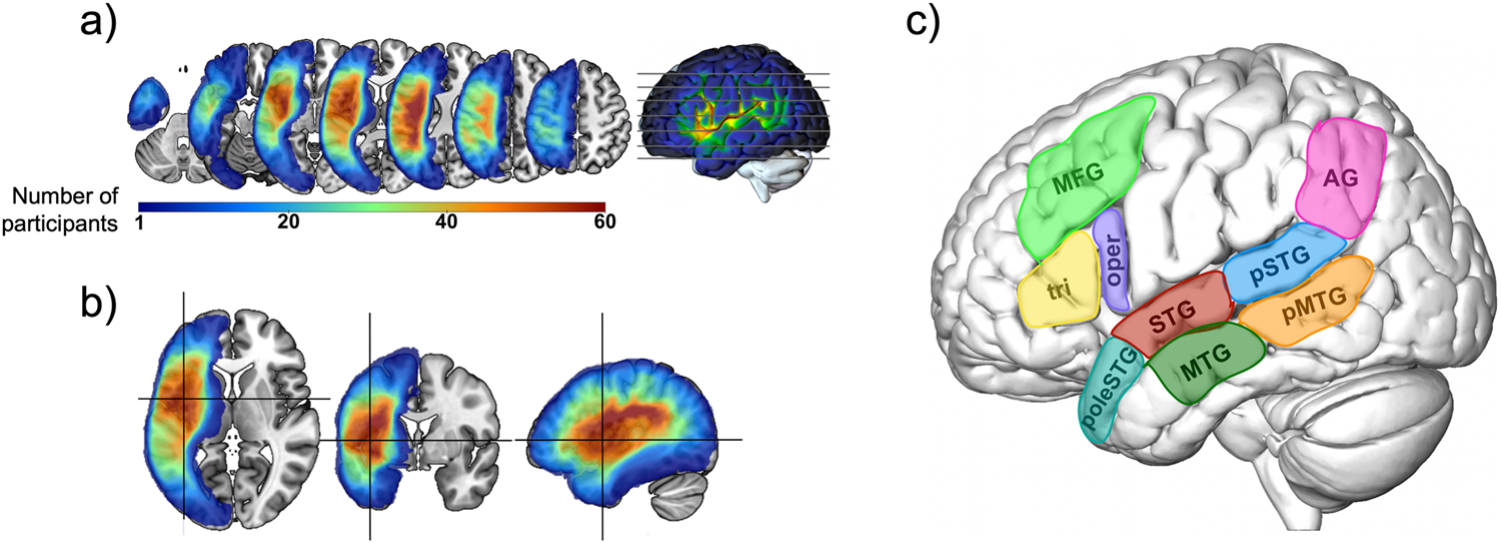
Lesion maps and pre-selected regions of interests. **a** Lesion overlay of participants with a stroke (*n* = 93) where different colors denote the number of participants having a lesion in that area, with warmer colors representing more participants having a lesion. **b** Peak of lesion overlay. The region with the highest number of participants (*n* = 70) presenting with a lesion was the rolandic operculum (−40.4 $\times$ −13.4 $\times$ 18.2; crosshair position). The scale for (**a**) is to be used for (**b**). **c** Visual schematic representation of pre-selected language-related regions of interests (MFG middle frontal gyrus, tri inferior frontal gyrus pars triangularis, oper inferior frontal gyrus pars *opercularis*, poleSTG pole of the superior temporal gyrus, STG superior temporal gyrus, pSTG posterior superior temporal gyrus, AG angular gyrus, MTG middle temporal gyrus, pMTG posterior middle temporal gyrus).

We studied data from participants with a stroke who were part of a randomized controlled clinical trial entitled POLAR (Predicting Outcome of Language Rehabilitation in Aphasia, clinicaltrials.gov ID: NCT03416738)^71^. The inclusion criteria of the POLAR trial specified that participants must have had a chronic ($\ge \!$12 months) unilateral stroke to the left hemisphere, be 21 to 80 years old, and speak English as their primary language for at least 20 years. Participants were excluded if they had bilateral or right-hemisphere strokes or other neurological brain-related illnesses. Participants with a stroke consisted of 93 individuals with left-hemisphere strokes primarily in middle cerebral artery territory (Fig. 5a, b). From 127 participants recruited for POLAR, we excluded 34 participants who were not diagnosed with aphasia or did not have the required MRI scans. By only including participants with aphasia following a left-hemisphere stroke, we assumed that all participants had left-hemisphere language dominance before their stroke. In the current study, we examined baseline data from the POLAR trial prior to treatment to test the relations among brain age, brain controllability, and aphasia severity.

Before study enrollment, all participants provided written informed consent. The POLAR clinical trial was conducted at the University of South Carolina (Columbia) and the Medical University of South Carolina (Charleston). Local Institutional Review Boards approved the study.

Of note, the original study^48^ that assessed the relation between brain age and aphasia also included the participants from POLAR, thus, there is an overlap between the study samples.

### Language assessment

Aphasia severity was determined using the Aphasia Quotient of the Western Aphasia Battery (Revised; WAB-AQ)^72^ before starting therapy (Table 1). Only participants with WAB-AQ scores of <93.7 were included, which is the cut-off value for diagnosing aphasia based on WAB-R criteria. Participants were excluded from the POLAR trial if they had severely impaired verbal output (i.e., a score of $\le$1 on the WAB-R spontaneous speech scale), or severely impaired auditory comprehension (i.e., a score of $\le$1 on the WAB-R comprehension scale).

**Table 1:** Demographic and diagnostic information of all participants (*n* = 93).

Demographic information		
Age (in years), mean (SD; range)		60.77 (11.19; 29–80)
Sex, *n* (%)	Female	37 (39.78)
	Male	56 (60.22)
Race, *n* (%)	Black or African American	22 (23.66)
	Asian	2 (2.15)
	Caucasian	69 (74.19)
Education (in years), mean (SD; range)		15.52 (2.33, 12–20)
Diagnostic information		
Months since stroke, mean (SD; range)		49.60 (52.51; 10–241)
Stroke lesion volume (in ml), mean (SD; range)		129.66 (96.60; 2.38–467.46)
WAB-AQ (max. 100), mean (SD; range)		58.77 (22.86; 14.50–93.10)

*n* number, *SD* standard deviation, *WAB-AQ* Aphasia Quotient of the Western Aphasia Battery (Revised) (Kertesz, 2007).

### Magnetic resonance image acquisition

All participants underwent structural MRI scanning including T1-weighted, T2-weighted, and diffusion weighted scans (diffusion tensor images, DTI). T2-weighted images were used to delineate the chronic stroke lesions. MRIs were acquired on a Siemens 3T Prisma scanner (Siemens Medical Systems, Erlangen, Germany) with a 20-channel head coil.

For theT1-weighted MR images we used an MPRAGE sequence with a resolution of 1 mm isotropic voxels, matrix size of 256 $\times$ 256, 9-degree flip angle, 192 slice sequence with repetition time of 2250 ms, inversion time of 925 ms, echo time of 4.11 ms, parallel imaging (GRAPPA = 2, 80 reference lines). For the T2-weighted MRI images we used a 3D turbo spin echo sequence with a matrix size of 256 × 256, variable flip angle, 176 1-mm-thick slices, repetition time of 3200 ms, echo time of 567 ms, parallel imaging (GRAPPA = 80 reference lines). For the DTI we used a monopolar echo planar imaging sequence with a matrix size of 140 $\times$ 140, 90-degree flip angle, sampling of 43 diffusion direction encodings (36 volumes with *b* = 1000 s/mm^2^, 7 volumes with *b* = 0 s/mm^2^), repetition time of 5250 ms, echo time of 80 ms, 210 $\times$ 210 mm^2^ field of view, parallel imaging GRAPPA = 2, 80 contiguous 1.5 mm thick slices. Phase encoding polarity was reversed for second acquisition of the same sequence.

**Fig. 6:**
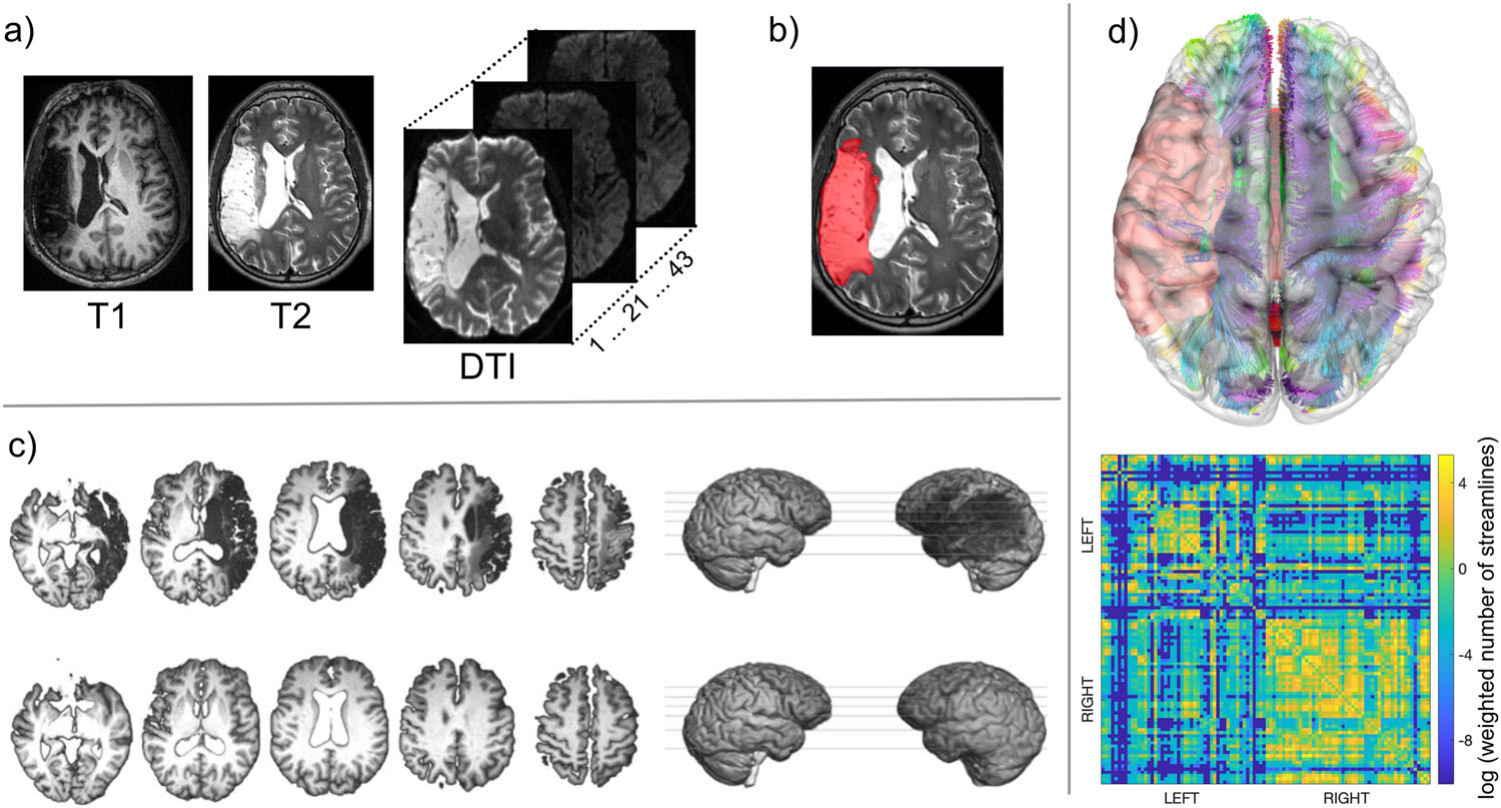
Image processing steps. **a** All participants underwent a structural brain MRI scan including T1, T2, and diffusion weighted images (DTI = diffusion tensor imaging) with 43 volumes. **b** Lesions were manually drawn on each participant’s T2-weighted image. **c** A chimeric T1 image was created for each participant where the stroke area was replaced by the mirrored equivalent of the intact, right hemisphere (healed T1, lower panel)^74^. The healed T1 was transformed into the Montreal Neurological Institute 152 (MNI152) standard space^75^. The stroke lesion was co-registered to the healed T1 standard space. Each participant’s T1-weighted image was segmented into gray matter regions of interest with the Johns Hopkins University anatomic atlas; the segmentation maps were registered into DTI space. **d** Probabilistic tractography was performed, and tracts were estimated between all pairs of gray matter regions. The procedure resulted in a 100 $\times$ 100 adjacency matrix where values represented the (corrected) number of probabilistic streamlines between two regions (heatmap in lower panel). Of note, the connectomes did not include the healed tissue. For better visualization, the color bars of the heatmaps represent log values, and the white matter connections in brain space (upper panel) are based on deterministic instead of probabilistic tractography.

All MR DICOM images were converted to NifTI format with the software dcm2niix^73^. MRI processing steps are shown in Fig. 6 and included lesion delineation and structural connectome processing.

### Lesion delineation

Lesions were manually drawn on T2-weighted images by a trained research specialist with supervision from LB, CR, or JF, using the software MRIcron (https://www.nitrc.org/projects/mricron) or MRIcroGL (https://www.nitrc.org/projects/mricrogl). The lesion maps in T2 space were co-registered to the participant’s T1 using in-house developed, open-source MATLAB scripts (https://github.com/neurolabusc/nii_preprocess)^74^ and SPM12 (Functional Imaging Laboratory, Wellcome Trust Center for Neuroimaging Institute of Neurology, University College London; http://www.fil.ion.ucl.ac.uk/spm/software/spm12/). Lesion maps in native T1 space were normalized into the Montreal Neurological Institute (MNI) 152 non-linear asymmetric standard brain template^75^. Normalization of the lesion maps was completed through the following steps: (1) smoothing of lesion maps by removing uneven edges with a 3-mm full-width at half-maximum Gaussian kernel, (2) binarizing of the smoothed lesion maps (lesioned vs. not lesioned tissue) with a threshold of 0; (3) enantiomorphic transformation of the participant’s T1-weighted image onto standard space^76^. In step 3, using the *nii_preprocess* pipeline (https://github.com/neurolabusc/nii_preprocess) and SPM12’s unified segmentation-normalization, a chimeric T1-weighted image was created with a voxel size = 1 mm^3^, where the stroke area was replaced by the mirrored equivalent of the intact, right hemisphere, to create chimeric images (i.e., ‘healed’ brains) to avoid tissue deformation^74^.

Total lesion volume was calculated as the sum of lesioned voxel (voxel size = 1 mm^3^) divided by 1000 to achieve units in ml. Total lesion volume served as a control variable in the statistical analyses explained below.

### Structural connectome processing

For every participant, we computed whole-brain probabilistic tractography structural connectomes using stroke-adapted connectome methods^14,46^. In brief, we segmented the normalized T1-weighted images into 100 gray matter regions of interest using the Johns Hopkins University (JHU) anatomic atlas^77^. We removed DTI distortions using eddy current corrections^78^ and registered the gray matter JHU maps to the DTI space. Next, we computed probabilistic tractography between every possible pair of gray matter regions using the probabilistic method of FSL’s FMRIB’s Diffusion Toolbox^79^ with the toolbox’s accelerated BEDPOST^80^ and probtrackX with 5000 individual pathways, drawn through probability distributions on principal fiber direction, curvature threshold = 0.2, maximum steps = 200, step length = 0.5 mm, and distance correction. We averaged the number of probabilistic streamlines B to A to obtain the weighted connectivity link for the pair of regions A and B. We corrected the connectivity links for the size of region A and the size of region B as well as the distance of the streamlines^81,82^. Each connectome consisted of a 100 $\times$ 100 matrix with nodes representing the 100 JHU gray matter regions and edges representing the weighted, undirected connectivity links. From this matrix, we excluded (i.e., set to zero) spurious links, which we defined as links whose weights were below the 20% percentile of the links in the right hemisphere^83^. We excluded brain regions from each participant’s connectome that were disconnected from the remaining network (e.g., due to the stroke), representing zero-degree nodes. The zero-degree nodes were excluded from the connectomes on a one-by-one basis resulting in a smaller adjacency matrix for participants with zero-degree nodes^21^. Regions that were damaged by the stroke lesion were included in the participants’ connectomes, if the region was at least partially connected to the remaining network (was not a zero-degree node).

### Brain age estimation

We adopted the brain age estimation pipeline brainageR (v2.1) from Cole and colleagues (github.com/james-cole/brainageR)^42,67–70,84^, which is a freely available software implemented in R using the kernlab package (R Core Team (2020). R: A language and environment for statistical computing. R Foundation for Statistical Computing, Vienna, Austria. URL https://www.R-project.org/). Brain-predicted age, henceforth referred to as brain age, is a voxel-wise estimate of regional volume covariance that has been established based on large cohorts for robustness for training and extrapolation purposes. Brain age is estimated based on raw structural T1-weighted images, which were segmented into gray matter, white matter, and cerebrospinal fluid using SPM12 (Fig. 6). Because our goal was to quantify brain health of the residual brain tissue, we used the chimeric T1 described above (where the stroke area was replaced by the mirrored homolog region in the right hemisphere) to exclude the stroke lesion from the brain age estimation for minimization of tissue distortion and measurement of brain age volume covariance based on non-lesioned tissue. Our recent work has demonstrated the feasibility, utility, and accuracy of the brain age estimation in a local population of individuals with strokes^47^.

Segmentation accuracy was verified through visual quality control. For normalization, the segmented images were registered to a custom template of the brain age pipeline and then affine registered into MNI152 space using non-linear spatial registration and SPM12’s DARTEL toolbox. The cerebrospinal fluid was removed, and the gray and white matter probabilistic tissues were vectorized, concatenated, and subjected to a principal component analysis to reduce dimensionality. The components that explained the top 80% of the variance were used for brain age prediction. A machine-learning algorithm using a pretrained Gaussian regression model implemented in R package Kernlab was used to estimate brain age for each participant using the coefficients from a full training model which has been validated previously. This model was trained to predict the chronological age of 3377 healthy individuals (comorbidities were excluded based on screenings) aged 18 to 92 years and tested on 857 healthy individuals. This model predicted chronological age with a mean absolute error of 3.93 years and explained 94.6% of the variance in chronological age. The model was tested on an entirely independent dataset including *N* = 611 healthy individuals aged 18–90 years. For this dataset, the model predicted chronological age with a mean absolute error of 4.90 years and explained 89.7% of the variance in chronological age. The model performance differed for younger compared to older individuals. On average, higher brain predicted than chronological ages were estimated for younger, and lower brain predicted than chronological ages were estimated for older individuals. Thus, controlling for chronological age in analyses using the brain age gap is recommended.

**Fig. 7:**
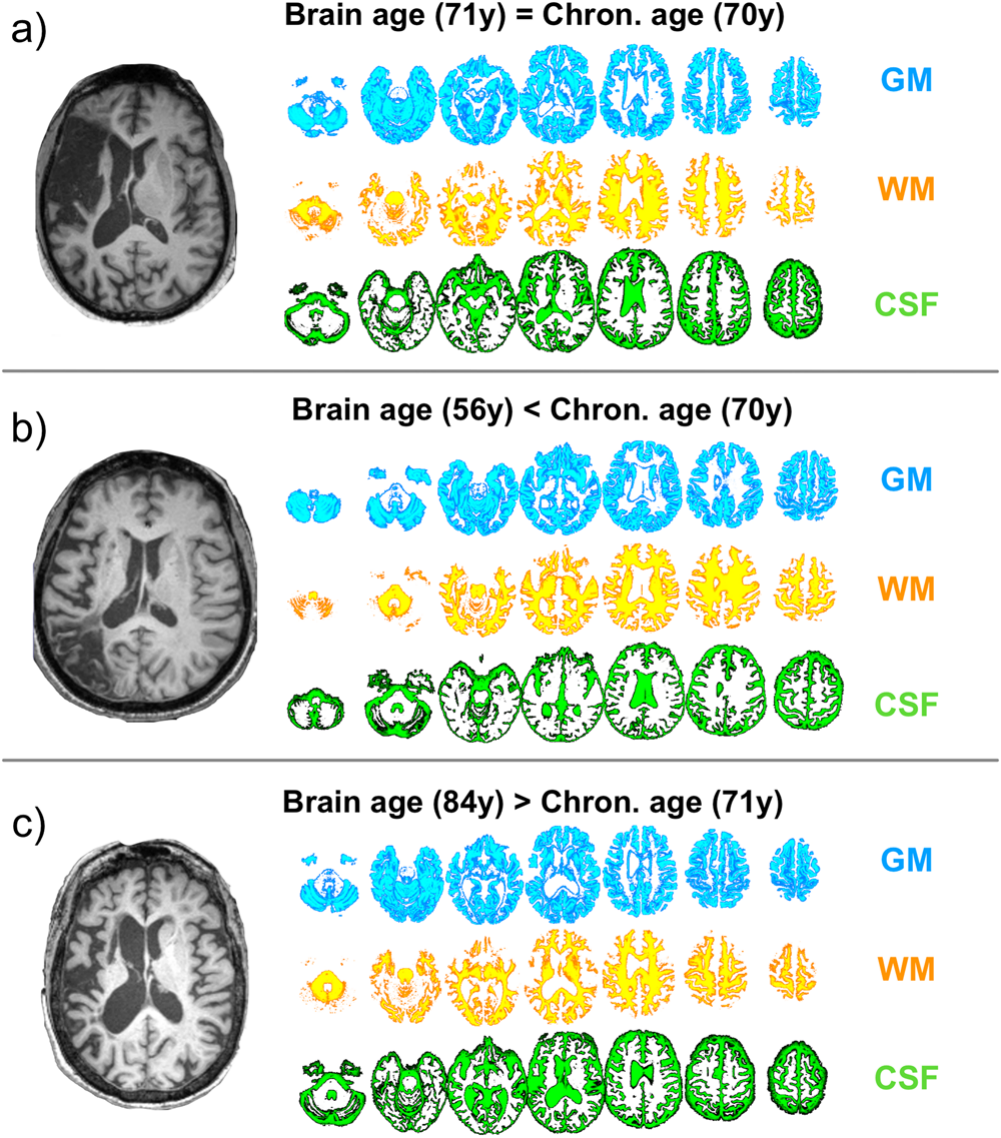
Brain age variability. Brain age was estimated from raw structural T1-weighted images, which were segmented into gray matter (GM), white matter (WM), and cerebrospinal fluid. **a**–**c** Three participants who had the same chronological age (70–71 years) but differed in their brain age. The brain age gap for participant A was 1 (=71–70), for participant B −14 (=56–70), and for participant C 13 (84–71). Of note, brain age was calculated from the healed T1 tissue to control for any impact of the stroke lesion on brain age estimates.

For each participant, we calculated brain age and the brain age gap, which refers to the difference between chronological and brain age (Fig. 7). Higher values of the brain age gap reflected older brain age than chronological age (i.e., advanced brain aging).

### Global network controllability

**Fig. 8:**
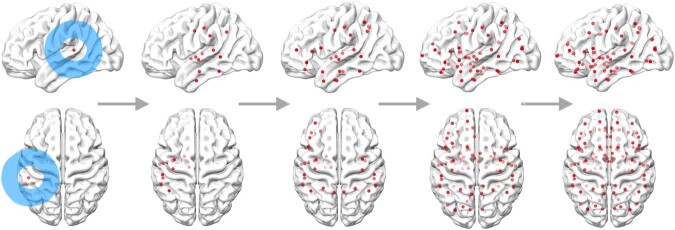
Schematic representation of network controllability. Input from a region in the network (blue circle) is distributed through the structural connections between nodes (red circles) and ultimately reaches all other nodes in the network. A network is controllable if any brain region can be influenced by input from all other regions.

The application of network control theory to neural systems is based on the assumptions that (1) brain function derives from brain structure, and (2) the brain is a dynamic system with regional activation patterns that change over time. Conceptually, different activation patterns represent different network states, which ultimately give rise to different functions and behaviors. Network controllability refers to the possibility of changing the network’s activation pattern and reaching a target state through the influence of a region within the network^24^. Brain networks are classified as controllable if brain states can be reached by the influence of each and all brain regions, which we term global controllability (Fig. 8).

Global controllability for a given network is computed from the smallest eigenvalue of the network’s controllability Gramian. If the smallest eigenvalue is >0, then the network is considered controllable^23,24^. Global network controllability is computed based on the network’s structural properties, captured in a connectome adjacency matrix *A*, where values represent the structural connectivity between two regions *i* and *j* (Fig. 6d). Previous studies with healthy individuals demonstrated that brain networks are controllable from any region^23,85^. However, a stroke may alter the brain network architecture so that a previously controllable network is not controllable after the stroke. Because zero-degree (disconnected) regions cannot control the remaining network’s activation patterns, we excluded all zero-degree nodes from the participants’ connectomes on a one-by-one basis resulting in a smaller adjacency matrix for these participants. While excluding zero-degree nodes resulted in controllable networks for most participants, some participants still did not have a controllable network. For example, if a region is connected to other regions (is not a zero-degree node), but these other regions are not connected to the remaining network, then this region cannot influence the activation state of the brain network and thus, the network is not globally controllable. Global controllability is a requirement for the valid application of average controllability.

### Average controllability

For each non-zero-degree gray matter region, we computed the ability to drive the network into a target state. Our previous research revealed that average controllability has the strongest relation with chronic post-stroke aphasia^21^. Thus, in the current study, we focused on average controllability and hypothesized that average controllability of language-specific regions mediates the relation between advanced brain age and aphasia severity.

Average controllability was calculated as *Trace(W*_*K*_*)*. Here W denotes the controllability Gramian of a connectome, and *K* denotes the set of nodes in the connectome. Average controllability is a measure of a node’s ability to spread and amplify energy through the entire network. The larger a node’s average controllability the better it can distribute energy to the other nodes in the system. In other words, average controllability is an approximation for the influence of a region over the activity of the remaining network^23,24^.

### Selection of language-related regions

We calculated the average controllability for a pre-selected set of 9 core brain regions in the left hemisphere that are involved in language processing. This set of regions spanned the left frontal to temporal and parietal areas and included the left middle frontal gyrus (posterior segment), inferior frontal gyrus (IFG) *pars opercularis* and *pars triangularis*, angular gyrus, superior temporal gyrus (STG), pole of STG, middle temporal gyrus, posterior STG, and posterior middle temporal gyrus (Fig. 5c). These regions are commonly referred to as language specific processing areas and are robustly linked to linguistic but not to non-linguistic tasks^86–88^. While many other brain areas are engaged in language processing, we focused on regions that are critical for language, as we hypothesized that these areas would have the strongest influence on the network dynamics of the language system, modeled as the regions’ average controllability.

**Fig. 9:**
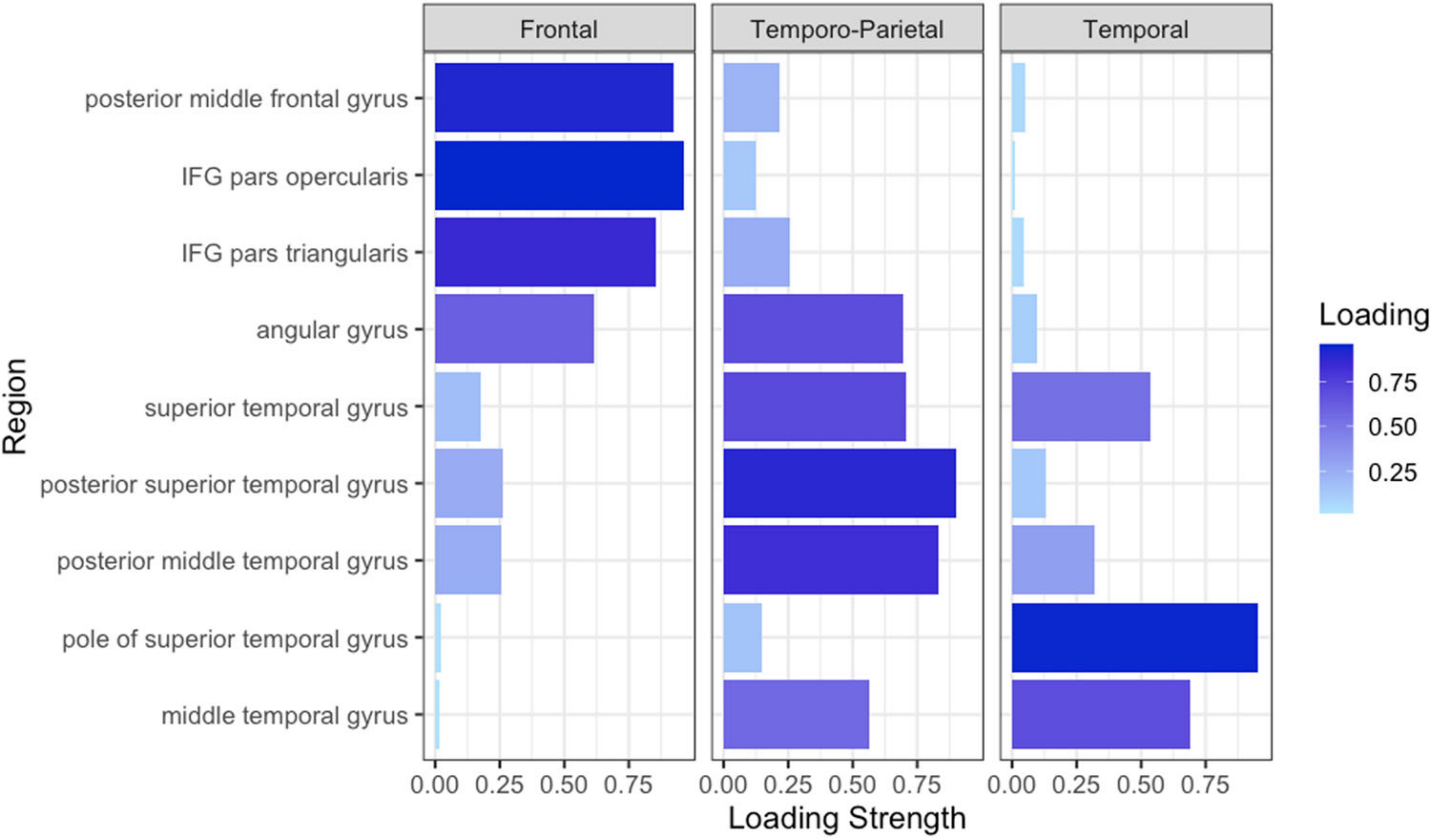
Results of principal component analysis. Each panel shows the principal component/factor loadings of each region’s average controllability on the factors named frontal, temporo-parietal, and temporal. Longer and more saturated bars indicate higher loadings. Graphical display adapted from https://rpubs.com/danmirman/plotting_factor_analysis.

Because of anatomical proximity, we assumed some degree of inter-correlation among the 9 regions’ average controllability values. Hence, we performed an explorative factor analysis (principal component analysis—PCA) to determine key dimensions of variability in the controllability of these 9 regions. Kaiser-Meyer-Olkin (KMO) and Bartlett’s test confirmed the suitability of our data for a PCA with values of >0.6 (0.765) for the KMO, and a significance level of <0.001 (*p* < 0.001 with 36 degrees of freedom) for the Bartlett’s test indicating the existence of a correlation across the variables and a data distribution that met the assumptions of multivariate analyses. A rotated component matrix (rotation converged in six iterations) was computed using PCA as the extraction (factors with an eigenvalue ≥1.0 were extracted) and varimax rotation with Kaiser Normalization as the rotation method. The PCA confirmed a three-factor solution, which accounted for 86.79% of variance in the patients’ performance (factor 1 = 56.24%; factor 2 = 22.73%; factor 3 = 7.82%). Figure 9 shows the factor loadings of each of the controllability components. The PCA revealed three main underlying brain areas: (1) a component associated with the controllability of frontal regions, (2) a component associated with temporo-parietal regions, and (3) a component associated with temporal regions. Among all 9 regions, the IFG *pars opercularis* had the strongest loading on factor 1 frontal (0.961), the posterior superior temporal gyrus on factor 2 temporo-parietal (0.899), and the pole of the superior temporal gyrus on factor 3 temporal (0.951). From here on, we will only report results for the IFG *pars opercularis*, posterior superior temporal gyrus, and pole of the superior temporal gyrus, because they best represented the three key dimensions, and because these ROIs are well established language regions.

### Statistics and reproducibility

We performed multiple linear regression modeling to assess the relation between the brain age gap (independent variable) and WAB-AQ (dependent variable) while controlling for chronological age, lesion volume, number of months since stroke, number of years of education, and sex. *P*-values < 0.05 were considered statistically significant. The WAB-AQ was chosen as the dependent variable based on its extensive use in aphasia research and clinical practice^89,90^ and its correlation with communication-related quality of life^91^. Further, our goal was to use a language measure that reflects the involvement of the broad neural network as estimated with the controllability measures employed in our study. We believe that the WAB-AQ is an appropriate tool for assessing this construct.

Mediation analysis was applied to assess the interplay among the brain age gap, average controllability, and WAB-AQ. Mediation analysis includes a combination of multiple linear regression models to draw conclusions for total, direct, and indirect effects among the independent, dependent, and mediating variables. In our study, the brain age gap was the independent variable, WAB-AQ the dependent variable, and average controllability the mediating variable. First, we tested for a significant total effect of the brain age gap on WAB-AQ using a regression model with brain age gap as the independent variable and with WAB-AQ as the dependent variable. After the total effect was established, we examined whether the brain age gap was directly or indirectly predictive of WAB-AQ. The direct effect was tested using multiple linear regression modeling with brain age gap as the independent variable and WAB-AQ as the dependent variable, while controlling for the mediating variable of average controllability. The indirect (mediating) effect was tested as the effect of brain age gap on WAB-AQ through average controllability. Here, we performed two regression models: (1) brain age gap as the independent variable, average controllability as the dependent variable, and (2) average controllability as the independent variable, WAB-AQ as the dependent variable. In all models, we controlled for lesion volume, chronological age, number of months since stroke, number of years of education, and sex.

We used model 4 of the PROCESS macro^92^ for SPSS (IBM SPSS Statistics for Windows (version 28, released 2021, IBM Corp., Armonk, N.Y., USA)) to compute the mediation models. We applied bias corrected bootstrapping with 5000 samples and 95% confidence intervals. The null hypothesis (no indirect effect present) was rejected if the confidence interval did not include zero. For the final model predicting WAB-AQ, we calculated the relative importance of each regressor for the WAB-AQ by using the Lindemann, Merenda and Gold (LMG) indices for R-squared decomposition implemented in the R package relaimpo, version 2.2-6^93^.

### Reporting summary

Further information on research design is available in the Nature Portfolio Reporting Summary linked to this article.

## Supplementary information

Supplementary Information

Description of Additional Supplementary Files

Supplementary Data 1

Reporting Summary

## Data Availability

Source data for Figs. 1, 2, 3 and 9 are provided in Supplementary Data 1. The conditions of our ethics approval do not permit public archiving of anonymized raw data. Data will be made available upon request to the corresponding author and in accordance with ethical procedures governing the reuse of sensitive data, which includes completion of a data sharing agreement and approval by the local ethics committee.

## Code availability

The code used for the MRI processing is available at http://www.fil.ion.ucl.ac.uk/spm/software/spm12/ and https://github.com/neurolabusc/nii_preprocess. The code used for calculating the brain age is available at http://www.github.com/james-cole/brainageR.
