# Supplementary material for: Dynamic network properties of the superior temporal gyrus mediate the impact of brain age gap on chronic aphasia severity
Source: Commun Biol. 2023 Jul 14;6:727. doi: 10.1038/s42003-023-05119-z (PMC10349039; doi:10.1038/s42003-023-05119-z)
Supplement: Supplementary file 1 — Supplementary Information [file 42003_2023_5119_MOESM1_ESM.pdf]

Supplementary material

for the article

“Dynamic network properties of the superior temporal gyrus mediate the impact of brain age gap on chronic aphasia severity”

Supplementary Figure 1: Regional lesion volume (in %) of the left inferior frontal gyrus pars opercularis (IFG pars opercularis), pole of the superior temporal gyrus (pole STG), and posterior superior temporal gyrus (posterior STG). a) Relation between the regional lesion volume of the IFG pars opercularis and posterior STG (Pearson correlation was not significant, p=0.56). b) Relation between the regional lesion volume of the pole STG and posterior STG (Pearson correlation was significant, p<0.001). c) Relation between the regional lesion volume of the IFG pars opercularis and pole STG (Pearson correlation was significant, p<0.001). d) Number of participants with no lesion (=0%) and with a lesion (>0%) to the IFG pars opercularis, pole STG, and posterior STG; and average (mean) and median regional lesion volume across all participants. The line the graphs a, b, c represents the fit line; the shading represents the 95% confidence interval.

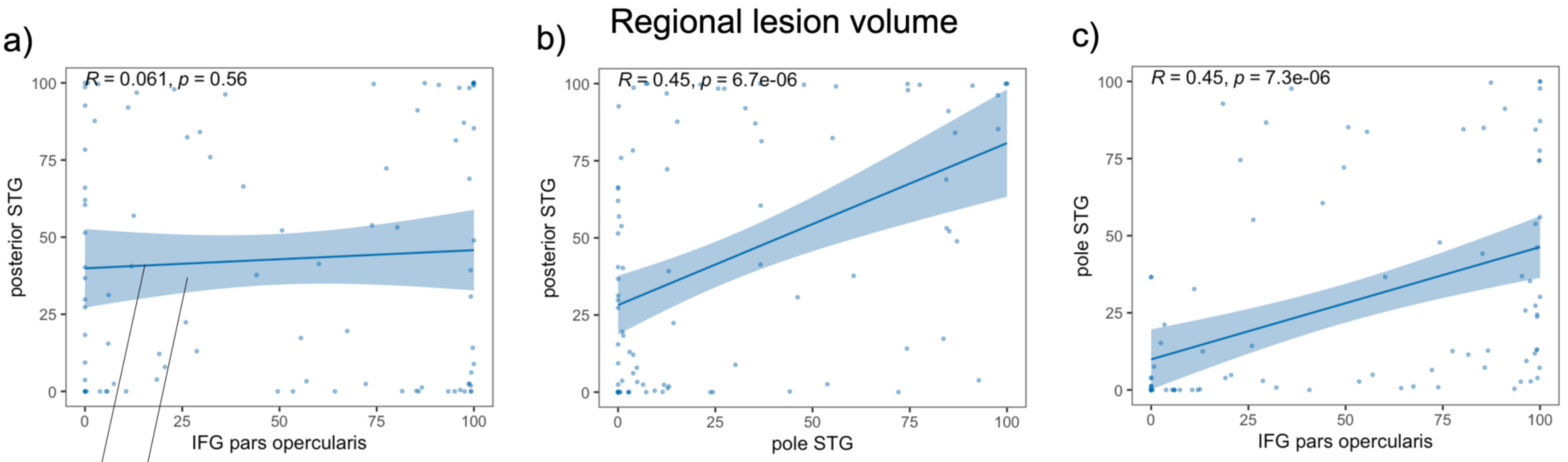

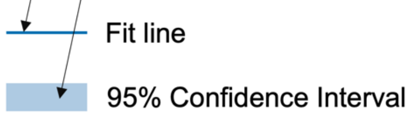

d)

	IFG pars opercularis	pole STG	posterior STG
**N (%) of participants with 0% lesion**	17 (18.28%)	21 (22.58%)	18 (19.35%)
**N (%) of participants with >0% lesion**	76 (81.72%)	72 (77.42%)	75 (80.65%)
**Average % lesion across participants**	48.88%	27.72%	42.77%
**Median % lesion across participants**	49.57%	11.45%	36.71%

Supplementary Table 1: Grey matter regions (n=100) included in each patient's connectome and displayed in the circular connectome diagrams. Regions on the left side of the circular diagrams correspond in counterclockwise order to regions listed from top to bottom in this table. Likewise, regions on the right side of the circular diagrams correspond in clockwise order to regions listed from top to bottom in this table. This table is adapted from Wilmskoetter et al. (2022) [1].

Region group in left hemisphere	Region from Johns Hopkins University anatomical atlas	Region group in right hemisphere
Left Frontal	superior frontal gyrus (posterior segment)	Right Frontal
	superior frontal gyrus (prefrontal cortex)	
	pole of superior frontal gyrus	
	middle frontal gyrus	
	middle frontal gyrus (dorsal prefrontal cortex)	
	inferior frontal gyrus pars opercularis	
	inferior frontal gyrus pars orbitralis	
	inferior frontal gyrus pars triangularis	
	lateral fronto-orbital gyrus	
	middle fronto-orbital gyrus	
	rectus gyrus	
	precentral gyrus	
	rostral anterior cingulate gyrus	
	subcallosal anterior cingulate gyrus	
	subgenual anterior cingulate gyrus	
	dorsal anterior cingulate gyrus	
Left Insula	anterior insula	Right Insula
	posterior insula	
Left Temporal	superior temporal gyrus	Right Temporal
	pole of superior temporal gyrus	
	middle temporal gyrus	
	pole of middle temporal gyrus	
	inferior temporal gyrus	
	parahippocampal gyrus	
	entorhinal area	
	fusiform gyrus	
	amygdala	
	hippocampus	
	posterior superior temporal gyrus	
	posterior middle temporal gyrus	
	posterior inferior temporal gyrus	
Left Subcortical	caudate nucleus	Right Subcortical

	putamen	
	globus pallidus	
	thalamus	
	hypothalamus	
	nucleus innominata of mynert	
	nucleus accumbens	
	mammillary body	
Left Parietal	postcentral gyrus	Right Parietal
	superior parietal gyrus	
	supramarginal gyrus	
	angular gyrus	
	pre-cuneus	
	posterior cingulate gyrus	
Left Occipital	superior occipital gyrus	Right Occipital
	middle occipital gyrus	
	inferior occipital gyrus	
	cuneus	
	lingual gyrus	

Supplementary References:

1. Wilmskoetter, J., He, X., Caciagli, L., Jensen, J. H., Marebwa, B., Davis, K. A., Fridriksson, J., Basilakos, A., Johnson, L. P., Rorden, C., Bassett, D., & Bonilha, L. (2022, Jan 26). Language Recovery after Brain Injury: A Structural Network Control Theory Study. Journal of Neuroscience, 42(4), 657-669.
