# Supplementary material for: Dynamic network properties of the superior temporal gyrus mediate the impact of brain age gap on chronic aphasia severity
Source: Commun Biol. 2023 Jul 14;6:727. doi: 10.1038/s42003-023-05119-z (PMC10349039; doi:10.1038/s42003-023-05119-z)
Supplement: Supplementary file 2 — Description of Additional Supplementary Files [file 42003_2023_5119_MOESM2_ESM.pdf]

Description of Additional Supplementary Files

File name: Supplementary Data 1
Description: Source Data for Figures 1, 2, 3, and 9
