# Supplementary material for: Dynamic network properties of the superior temporal gyrus mediate the impact of brain age gap on chronic aphasia severity
Source: Commun Biol. 2023 Jul 14;6:727. doi: 10.1038/s42003-023-05119-z (PMC10349039; doi:10.1038/s42003-023-05119-z)
Supplement: Supplementary file 4 — Reporting Summary [file 42003_2023_5119_MOESM4_ESM.pdf]

nature portfolio

Corresponding author(s): Janina Wilmskoetter

Last updated by author(s): 07/03/2023



# Reporting Summary

Nature Portfolio wishes to improve the reproducibility of the work that we publish. This form provides structure for consistency and transparency in reporting. For further information on Nature Portfolio policies, see our Editorial Policies and the Editorial Policy Checklist.

## Statistics

For all statistical analyses, confirm that the following items are present in the figure legend, table legend, main text, or Methods section.

| n/a | Confirmed | |
|---|---|---|
| ☐ | ☒ | The exact sample size (*n*) for each experimental group/condition, given as a discrete number and unit of measurement |
| ☐ | ☒ | A statement on whether measurements were taken from distinct samples or whether the same sample was measured repeatedly |
| ☐ | ☒ | The statistical test(s) used AND whether they are one- or two-sided<br>*Only common tests should be described solely by name; describe more complex techniques in the Methods section.* |
| ☐ | ☒ | A description of all covariates tested |
| ☐ | ☒ | A description of any assumptions or corrections, such as tests of normality and adjustment for multiple comparisons |
| ☐ | ☒ | A full description of the statistical parameters including central tendency (e.g. means) or other basic estimates (e.g. regression coefficient) AND variation (e.g. standard deviation) or associated estimates of uncertainty (e.g. confidence intervals) |
| ☐ | ☒ | For null hypothesis testing, the test statistic (e.g. *F*, *t*, *r*) with confidence intervals, effect sizes, degrees of freedom and *P* value noted<br>*Give P values as exact values whenever suitable.* |
| ☐ | ☒ | For Bayesian analysis, information on the choice of priors and Markov chain Monte Carlo settings |
| ☐ | ☒ | For hierarchical and complex designs, identification of the appropriate level for tests and full reporting of outcomes |
| ☐ | ☒ | Estimates of effect sizes (e.g. Cohen's *d*, Pearson's *r*), indicating how they were calculated |

*Our web collection on statistics for biologists contains articles on many of the points above.*

## Software and code

Policy information about availability of computer code

| | |
|---|---|
| Data collection | NA |
| Data analysis | Lesions were drawn with MRIcron (https://www.nitrc.org/projects/mricron) or MRIcroGL (https://www.nitrc.org/projects/mricrogl). The lesion maps in T2 space were co-registered to the participant's T1 using in-house developed, opensource MATLAB scripts (https://github.com/neurolabusc/nii_preprocess) and SPM12 (Functional Imaging Laboratory, Wellcome Trust Center for Neuroimaging Institute of Neurology, University College London; http://www.fil.ion.ucl.ac.uk/spm/software/spm12/). using the nii_preprocess pipeline (https://github.com/neurolabusc/nii_preprocess) and SPM12's unified segmentation-normalization, a chimeric T1-weighted image was created. We adopted the brain age estimation pipeline brainageR (v2.1) from Cole and colleagues (github.com/james-cole/brainageR) 45,64-68, which is a freely available software implemented in R using the kernlab package (R Core Team (2020). R: A language and environment for statistical computing. R Foundation for Statistical Computing, Vienna, Austria. URL https://www.R-project.org/). |

For manuscripts utilizing custom algorithms or software that are central to the research but not yet described in published literature, software must be made available to editors and reviewers. We strongly encourage code deposition in a community repository (e.g. GitHub). See the Nature Portfolio guidelines for submitting code & software for further information.

## Data

Policy information about availability of data

All manuscripts must include a data availability statement. This statement should provide the following information, where applicable:

- Accession codes, unique identifiers, or web links for publicly available datasets
- A description of any restrictions on data availability
- For clinical datasets or third party data, please ensure that the statement adheres to our policy

The conditions of our ethics approval do not permit public archiving of anonymized raw data. Data will be made available upon request to the corresponding author and in accordance with ethical procedures governing the reuse of sensitive data, which includes completion of a data sharing agreement and approval by the local ethics committee.

## Human research participants

Policy information about studies involving human research participants and Sex and Gender in Research.

| | |
|---|---|
| Reporting on sex and gender | Sex (self-reported by participants): 37 females (39.78%), 56 males (60.22%). We controlled for sex in our statistical analyses. |
| Population characteristics | Age (in years), mean (SD; range): 60.77 (11.19; 29–80)<br>Sex, n (%): Females: 37 (39.78), Males: 56 (60.22)<br>Race, n (%): Black or African American :22 (23.66), Asian: 2 (2.15), Caucasian: 69 (74.19)<br>Education (in years), mean (SD; range): 15.52 (2.33, 12–20)<br>Months since stroke, mean (SD; range): 49.60 (52.51; 10–241)<br>Stroke lesion volume (in ml), mean (SD; range): 129.66 (96.60; 2.38–467.46)<br>Western Aphasia Battery - Aphasia Quotient (max. 100), mean (SD; range): 58.77 (22.86; 14.50–93.10) |
| Recruitment | We studied data from participants with a stroke who were part of a randomized controlled clinical trial entitled POLAR (Predicting Outcome of Language Rehabilitation in Aphasia, clinicaltrials.gov ID: NCT03416738) 71. The inclusion criteria of the POLAR trial specified that participants must have had a chronic (≥12 months) unilateral stroke to the left hemisphere, be 21 to 80 years old, and speak English as their primary language for at least 20 years. Participants were excluded if they had bilateral or right-hemisphere strokes or other neurological brain-related illnesses. Participants with a stroke consisted of 93 individuals with left-hemisphere strokes primarily in middle cerebral artery territory (Figures 5a, 5b). From 127 participants recruited for POLAR, we excluded 34 participants who were not diagnosed with aphasia or did not have the required MRI scans. |
| Ethics oversight | Before study enrollment, all participants provided written informed consent. The POLAR clinical trial was conducted at the University of South Carolina (Columbia) and the Medical University of South Carolina (Charleston). Local Institutional Review Boards approved the study. |

Note that full information on the approval of the study protocol must also be provided in the manuscript.

# Field-specific reporting

Please select the one below that is the best fit for your research. If you are not sure, read the appropriate sections before making your selection.

☐ Life sciences ☒ Behavioural & social sciences ☐ Ecological, evolutionary & environmental sciences

For a reference copy of the document with all sections, see nature.com/documents/nr-reporting-summary-flat.pdf

# Behavioural & social sciences study design

All studies must disclose on these points even when the disclosure is negative.

| | |
|---|---|
| Study description | Quantitative experimental study (We studied data from participants with a stroke who were part of a randomized controlled clinical trial ). |
| Research sample | From 127 participants recruited for the RCT POLAR, we included 93 participants who were diagnosed with aphasia and had the required MRI scans. By only including participants with aphasia following a left-hemisphere stroke, we assumed that all participants had left-hemisphere language dominance before their stroke. |
| Sampling strategy | See previous answer for how we derived the sample size. |
| Data collection | Data were collected with computer and MRI machines. |

| | |
|---|---|
| Timing | The POLAR RCT ran from 2016 to 2021. From 127 participants recruited for POLAR, we excluded 34 participants who were not diagnosed with aphasia or did not have the required MRI scans. |
| Data exclusions | From 127 participants recruited for POLAR, we excluded 34 participants who were not diagnosed with aphasia or did not have the required MRI scans. |
| Non-participation | NA. We leveraged data from a clinical trial. |
| Randomization | In the current study, we examined baseline data from the POLAR trial prior to treatment randomization. |

# Reporting for specific materials, systems and methods

We require information from authors about some types of materials, experimental systems and methods used in many studies. Here, indicate whether each material, system or method listed is relevant to your study. If you are not sure if a list item applies to your research, read the appropriate section before selecting a response.

## Materials & experimental systems

| n/a | Involved in the study | |
|---|---|---|
| ☒ | ☐ | Antibodies |
| ☒ | ☐ | Eukaryotic cell lines |
| ☒ | ☐ | Palaeontology and archaeology |
| ☒ | ☐ | Animals and other organisms |
| ☐ | ☒ | Clinical data |
| ☒ | ☐ | Dual use research of concern |

## Methods

| n/a | Involved in the study | |
|---|---|---|
| ☒ | ☐ | ChIP-seq |
| ☒ | ☐ | Flow cytometry |
| ☐ | ☒ | MRI-based neuroimaging |

## Clinical data

Policy information about clinical studies

All manuscripts should comply with the ICMJE guidelines for publication of clinical research and a completed CONSORT checklist must be included with all submissions.

| | |
|---|---|
| Clinical trial registration | POLAR (Predicting Outcome of Language Rehabilitation in Aphasia, clinicaltrials.gov ID: NCT03416738) |
| Study protocol | https://clinicaltrials.gov/ct2/show/NCT03416738 |
| Data collection | Data collection was conducted between 2016 and 2021 at the University of South Carolina and the Medical University of South Carolina. |
| Outcomes | For the clinical trial, the primary outcome measure was the Philadelphia Naming Test [Time Frame: 6 months] to assesses the ability to name functional objects. Of note, in our study we only used baseline data. and used the Western Aphasia Battery (Revised; WAB-AQ) as the outcome measure to assess aphasia severity. |

## Magnetic resonance imaging

### Experimental design

| | |
|---|---|
| Design type | Structural MRI |
| Design specifications | NA - no fMRI used in this study. |
| Behavioral performance measures | NA |

### Acquisition

| | |
|---|---|
| Imaging type(s) | Structural MRI: T1, T2, DTI |
| Field strength | 3T |
| Sequence & imaging parameters | T1-weighted images: MPRAGE sequence with a resolution of 1mm isotropic voxels, matrix size of 256×256, 9-degree flip angle, 192 slice sequence with repetition time of 2250 ms, inversion time of 925 ms, echo time of 4.11 ms, parallel imaging (GRAPPA = 2, 80 reference lines).<br>T2-weighted images: 3D turbo spin echo sequence with a matrix size of 256x256, variable flip angle, 176 1-mm thick slices, repetition time of 3200 ms, echo time of 567 ms, parallel imaging (GRAPPA = 80 reference lines). |
| Area of acquisition | Whole brain |

Diffusion MRI ☒ Used ☐ Not used

Parameters: DTI: monopolar echo planar imaging sequence with a matrix size of 140×140, 90-degree flip angle, sampling of 43 diffusion direction encodings (36 volumes with b = 1000 s/mm2, 7 volumes with b = 0 s/mm2), repetition time of 5250 ms, echo time of 80 ms, 210×210 mm2 field of view, parallel imaging GRAPPA = 2, 80 contiguous 1.5mm thick slices. Phase encoding polarity was reversed for second acquisition of the same sequence.

## Preprocessing

Preprocessing software: nii_preprocess pipeline (https://github.com/neurolabusc/nii_preprocess)

Normalization: Lesion maps in native T1 space were normalized into the Montreal Neurological Institute (MNI) 152 non-linear asymmetric standard brain template 55. Normalization of the lesion maps was completed through the following steps: (1) smoothing of lesion maps by removing uneven edges with a 3-mm full-width at half-maximum Gaussian kernel, (2) binarizing of the smoothed lesion maps (lesioned vs. not lesioned tissue) with a threshold of 0; (3) enantiomorphic transformation of the participant's T1-weighted image onto standard space 56. In step 3, using the nii_preprocess pipeline (https://github.com/neurolabusc/nii_preprocess) and SPM12's unified segmentation-normalization, a chimeric T1-weighted image was created with a voxel size = 1 mm3, where the stroke area was replaced by the mirrored equivalent of the intact, right hemisphere, to create chimeric images (i.e., 'healed' brains) to avoid tissue deformation.

Normalization template: Native T1 space were normalized into the Montreal Neurological Institute (MNI) 152 non-linear asymmetric standard brain template.

Noise and artifact removal: Normalization of the lesion maps was completed through the following steps: (1) smoothing of lesion maps by removing uneven edges with a 3-mm full-width at half-maximum Gaussian kernel, (2) binarizing of the smoothed lesion maps (lesioned vs. not lesioned tissue) with a threshold of 0; (3) enantiomorphic transformation of the participant's T1-weighted image onto standard space.

Volume censoring: NA

## Statistical modeling & inference

Model type and settings: We performed multiple linear regression modeling to assess the relation between the brain age gap (independent variable) and WAB-AQ (dependent variable) while controlling for chronological age, lesion volume, number of months since stroke, number of years of education, and sex. P-values <0.05 were considered statistically significant. Mediation analysis was applied to assess the interplay among the brain age gap, average controllability, and WAB-AQ.

Effect(s) tested: See prior response

Specify type of analysis: ☐ Whole brain ☐ ROI-based ☒ Both

Anatomical location(s): We segmented the normalized T1-weighted images into 100 gray matter regions of interest using the Johns Hopkins University anatomic atlas.

Statistic type for inference (See Eklund et al. 2016): NA

Correction: NA

## Models & analysis

| n/a | Involved in the study |
|---|---|
| ☒ | ☐ Functional and/or effective connectivity |
| ☐ | ☒ Graph analysis |
| ☐ | ☒ Multivariate modeling or predictive analysis |

Graph analysis: Average controllability was calculated as Trace(WK). Here W denotes the controllability Gramian of a connectome, and K denotes the set of nodes in the connectome. Average controllability is a measure of a node's ability to spread and amplify energy through the entire network. The larger a node's average controllability the better it can distribute energy to the other nodes in the system. In other words, average controllability is an approximation for the influence of a region over the activity of the remaining network.

Multivariate modeling and predictive analysis: We performed multiple linear regression modeling to assess the relation between the brain age gap (independent variable) and WAB-AQ (dependent variable) while controlling for chronological age, lesion volume, number of months since stroke, number of years of education, and sex. P-values <0.05 were considered statistically significant.
Mediation analysis was applied to assess the interplay among the brain age gap, average controllability, and WAB-AQ. Mediation analysis includes a combination of multiple linear regression models to draw conclusions for total, direct, and indirect effects among the independent, dependent, and mediating variables. In our study, the brain age gap was the independent variable, WAB-AQ the dependent variable, and average controllability the mediating variable. First, we tested for a significant total effect of the brain age gap on WAB-AQ using a regression model with brain age gap as the independent variable and with WAB-

AQ as the dependent variable. After the total effect was established, we examined whether the brain age gap was directly or indirectly predictive of WAB-AQ. The direct effect was tested using multiple linear regression modeling with brain age gap as the independent variable and WAB-AQ as the dependent variable, while controlling for the mediating variable of average controllability. The indirect (mediating) effect was tested as the effect of brain age gap on WAB-AQ through average controllability. Here, we performed two regression models: 1) brain age gap as the independent variable, average controllability as the dependent variable, and 2) average controllability as the independent variable, WAB-AQ as the dependent variable. In all models, we controlled for lesion volume, chronological age, number of months since stroke, number of years of education, and sex.
We used model 4 of the PROCESS macro for SPSS (IBM SPSS Statistics for Windows (version 28, released 2021, IBM Corp., Armonk, N.Y., USA)) to compute the mediation models. We applied bias corrected bootstrapping with 5000 samples and 95% confidence intervals. The null hypothesis (no indirect effect present) was rejected if the confidence interval did not include zero. For the final model predicting WAB-AQ, we calculated the relative importance of each regressor for the WAB-AQ by using the Lindemann, Merenda and Gold (LMG) indices for R-squared decomposition implemented in the R package relaimpo, version 2.2-6.
